# Immunity after Vaccination against COVID-19

**DOI:** 10.3390/vaccines11111723

**Published:** 2023-11-17

**Authors:** Leszek Tylicki

**Affiliations:** Department of Nephrology, Transplantology and Internal Medicine, Faculty of Medicine, Medical University of Gdańsk, 80-210 Gdańsk, Poland; leszek.tylicki@gumed.edu.pl

The outbreak of the COVID-19 pandemic at the turn of 2019 and 2020 posed a substantial challenge for the world. The number of infected people and deaths reached unprecedented levels, paralyzing health services in many countries [1]. Vaccination was the most effective tool to control the pandemic. In clinical practice, decreased infections, a milder course of COVID-19, and a reduced mortality rate were visible after vaccinations [2]. Nevertheless, the following remains an open question: To what extent did natural immunity (pre-vaccinations) contribute to these numbers? We should also remember that the natural course of the pandemic and subsequent mutations of the SARS-CoV-2 weakened the virus and led to a milder course of the disease and the gradual extinction of the pandemic [3]. Regardless of the above considerations, vaccination remains the only option that effectively protects against primary infection. Despite the efforts of the scientific world and vaccination of a large part of the world’s population, many aspects of the immune response against COVID-19 remain unclear. This Special Issue of *Vaccines* is a platform where scientists from different countries can present the current state of knowledge and the results of their studies in this area. Eleven interesting articles, including two review papers, were published there.

In a fascinating overview, Kaminska et al. (Contribution 1) presented the current knowledge on immunity after SARS-CoV-2 infection and vaccination against COVID-19. The authors also discussed the diagnostic and research tools available to examine the anti-SARS-CoV-2 cellular and humoral immune responses. In general, the immune responses generated after SARS-CoV-2 infection or vaccination seem similar. However, they differ in the details, e.g., higher post-vaccination antibody titers and a somewhat longer duration of post-infectious response. In addition, infection induces immune responses to a broader array of viral antigens. SARS-CoV-2 induces both humoral and cellular immune responses against spike (S), membrane (M), and nucleocapsid (N) antigens [4]. Clinically, infection was found to provide higher protection against reinfection and more sustained protection against hospital admission or severe disease than vaccination alone [5].

The adaptive immune response consists of two complementary branches: humoral and cellular T cell-mediated immunity. Cellular immunity is less understood, largely due to a lack of validated diagnostic tests. T cells, with their diverse set of receptors, seem to have the advantage of recognizing a wider range of epitopes displayed in infected or antigen-presenting cells as being either an MHC class I or II surface protein. In the context of SARS-CoV-2 infection, while antibodies can protect us from developing an infection, T lymphocytes prevent the disease from becoming severe by eliminating infected cells [6]. Primorac et al. (Contribution 2) presented interesting results of their original study on cellular immunity. They showed that cellular immunity, determined by measuring interferon-gamma levels, provided long-term protection against SARS-CoV-2. At the same time, measurements of humoral immunity (antibody levels) decreased over time. The level of cellular immunity in the vaccinated patients was equal to that of study participants previously infected with SARS-CoV-2. 

Two other papers are devoted to hybrid immunity against COVID-19, obtained via vaccination and SARS-CoV-2 infection at any order (Contributions 3 and 4). Nicola Serra et al. retrospectively evaluated antibody responses in a sample of 538 healthcare workers with a documented complete immunization cycle of three doses of mRNA vaccine against COVID-19 via multiplex assays. They found that patients who had also a history of asymptomatic SARS-CoV-2 infection had the highest titers of the anti-RBD and anti-S1 antibodies (Contribution 3). Blaszczuk et al. (Contribution 5) reported that individual SARS-CoV-2 variants can induce an immune response of varying strength in patients previously vaccinated against COVID-19, specifically the level of antibodies after SARS CoV-2 Omicron variant infection was lower than after Delta infection. A history of COVID-19 is associated with much stronger humoral immunity observed after vaccination in immunocompromised individuals [7]. Hybrid immunity seems not only robust but also more durable than either natural immunity alone or vaccine immunity alone. The longer duration of antigen expression following natural infection compared with mRNA vaccination may be related to the greater durability of immune memory [8]. Hybrid immunization also improves the functional antibody response, especially in those who have received the vector vaccine [9]. It is, therefore, not surprising that real-life efficacy data indicate that vaccination after prior SARS-CoV2 infection provides the highest level of protection against severe COVID-19 disease [10]. This is also in line with the results of the latest meta-analysis on this issue [5]. One can assume that the hybrid immunity that a large part of the world’s population had, combined with the attenuated viral pathogenicity of the Omicron variant prevalent, considerably reduced COVID-19 hospitalization and mortality compared with the early phase of the pandemic achieved in 2022 and 2023. 

COVID-19 is a threat to immunocompromised individuals, owing to their impaired natural immunity and suboptimal response to vaccines or immunosuppressive treatment. Consequently, they are at increased risk of hospitalization and death. These include subjects with primary immunodeficiency, end-stage renal disease patients, solid organ transplants, and subjects with solid tumors, among others [11]. For instance, the mortality rate of chronically hemodialyzed patients in the early period of the COVID-19 pandemic before vaccination was close to 31% of the total infected subjects; among patients over 75 years of age, it was close to 44% and appeared nearly 5.5 times higher than in the general population [12]. Several papers published in this Special Issue dealt with the clinical experience of COVID-19 vaccination in immunocompromised individuals. A reduced humoral response after receiving a second or third mRNA vaccine dose compared to healthy individuals was demonstrated in chronically hemodialyzed patients, solid organ transplant recipients, subjects with prostate cancer, pediatric patients with inflammatory bowel disease receiving anti-TNFα therapies (infliximab or adalimumab) (Contributions 6–9). The results of these studies also indicate that the degree of impairment of the post-vaccination immune response varies between these populations. Thus, only 23.5% of solid organ transplant recipients treated with belatacept developed a detectable anti-spike response after three doses of the BNT162b2 mRNA COVID-19 vaccine (Contribution 9). In contrast, the humoral response in hemodialyzed patients after the third dose of mRNA COVID-19 vaccine was very good, raising the level of antibodies to a higher level than in subjects from the general population who received the primary two-dose scheme of vaccination (Contribution 8). This indicates the need for an individualized approach to the primary and boosting vaccination schedule in different immunocompromised populations and the need to monitor immunological responses [13]. Khong et al. present interesting findings in their overview paper in which the benefits of a fourth booster dose are evaluated from four perspectives, including the effectiveness of the booster dose against virus variants (why), susceptible groups of individuals who may benefit from additional booster dose (who), selection of vaccine platforms to better enhance immunity (what), and appropriate intervals between the third and fourth booster dose (when) (Contribution 11). A fourth dose can be considered for certain groups of individuals, such as older people, the immunocompromised, and previous vaccine platforms. A heterologous vaccine strategy using an mRNA-based platform in subjects primed with inactivated vaccines may boost immunity against variants. The timing of the fourth dose should be individualized, but a 4-month interval after the third booster shot seems appropriate. The results of the studies presented in our Special Issue also confirm the validity of administering complementary vaccine doses (going beyond the basic scheme) to those immunocompromised individuals who did not respond to previous doses of the vaccine or whose humoral responses were too small (Contributions 8 and 10).

Although many aspects of COVID-19 immunity and vaccine response have already been clarified, there is still a sizable gap between clinicians’ questions and the available explanations, especially given the evolution of SARS-CoV-2 and new emerging variants. This will certainly be a subject of further research.
